# The Middle-Income Trap and the Coping Strategies From Network-Based Perspectives

**DOI:** 10.3390/e20100803

**Published:** 2018-10-18

**Authors:** Ming-Yang Zhou, Wen-Man Xiong, Xiao-Yu Li, Hao Liao

**Affiliations:** Guangdong Province Key Laboratory of Popular High Performance Computers, College of Computer Science and Software Engineering, Shenzhen University, Shenzhen 518060, China

**Keywords:** fitness, complexity, economics, middle-income trap, complex network

## Abstract

When a developing country reaches a relatively average income level, it often stops growing further and its income does not improve. This is known as the middle-income trap. How to overcome this trap is a longstanding problem for developing countries, and has been studied in various research fields. In this work, we use the Fitness-Complexity method (FCM) to analyze the common characteristics of the countries that successfully get through the middle-income trap, and show the origin of the middle-income trap based on the international trade network. In the analysis, a novel method is proposed to characterize the interdependency between products. The results show that some middle-complexity products depend much on each other, which indicates that developing countries should focus on them simultaneously, implying high difficulty to escape the middle-income trap. To tackle the middle-income trap, developing countries should learn experiences from developed countries that share similar development history. we then design an effective method to evaluate the similarity between countries and recommend developed countries to a certain developing country. The effectiveness of our method is validated in the international trade network.

## 1. Introduction

How does the economy of a country grow? Where does the wealth of a country come from? Does a developing country has potential to become a developed country and how should the country upgrade its industrial structure? Finding the solutions to these complicated questions and discovering the underlying rules that drive the economy is a longstanding problem in the field of economics [1,2,3,4]. In real scenarios, developed countries export some high-tech and profitable products [5,6], since the high-tech products help maintain and improve their competitiveness, while developing countries only produce some low-complexity products. If a developing country desires to improve its competitiveness, there is no doubt that the country should update its industrial structure. However, a disputed issue arises: whether the country should shift its attention from low-complexity to high-complexity products. The classical economic theories of Ricardo emphasize the importance of specialization on few high-complexity products [5], whereas recent research based on the complex network model shows that developed countries tend to diversify their export basket to improve their competitiveness [3,7], meaning that competitive countries have better industrial structure on both high- and low-complexity products.

Indeed, every developing country wants to improve its domestic economy and competitiveness. Empirical data show that, when the per capita GDP of developing countries reaches around $1000, the GDP per capita will sharply increase to $3000, known as the economic take-off phase [8]. (Pugliese et al. [9] further showed that the take-off phase could be precisely described by the fitness of countries.) However, during the period of $3000–$12,000 (the exact range standard may fluctuate a little), a large amount of domestic problems occur simultaneously, which hinders the further development of the countries, i.e., middle-income trap [10,11]. If the developing countries successfully overcome this economic bottleneck, they will step into the group of developed countries. The classical successful countries include the Asian tigers (South Korea, Singapore, Hong Kong and Taiwan), Japan, Germany, Italy and Spain. Some countries still suffer from the middle-income trap, including Argentina, Mexico and Brazil. It seems that there is no standard method and path to get through the trap. Nevertheless, the experience of the successful countries can help developing countries avoid many confusing economic problems [12,13]. However, different developed countries may have different, or even competing, policies. Thus, how to recommend an appropriate developed country to follow is also a challenging problem for developing countries [14,15]. Intuitively, a developing country should follow the developed countries that have similar economic background and history. The central problem is the evaluation of the economic similarity between countries. To characterize the similarity, classical methods usually require many economic data regarding industrial structure, investment, education, etc. which are unavailable in most cases. Network-based theory was introduced into the economic complexity to overcome this limitation, as it only requires few data, yet has high precision. With a network-based method, we only determine whether a country exports some certain products, where the export of a country could be extracted from the public international trade dataset [16]. There are two main network-based methods: (1) the Method of Refections (MR) uses a linear iterative process to upgrade the scores of countries and products, which is similar to PageRank [17,18,19]; and (2) the Fitness-Complexity Method (FCM) uses a nonlinear iterative method to calculate the fitness of countries and complexity of products respectively [3]. Both methods outperform previous governance, education and other economic competitiveness indices [20,21] and have been applied to other fields [22,23]. Since FCM predicts the economic growth with very high precision, it has attracted much attention and has been reported in Nature news (http://www.nature.com/news/physicists-make-weather-forecasts-for-economies-1.16963). However, the problem of how to help a country to find suitable developed countries to follow still lacks in-depth investigation.

In this paper, we first explore the characteristics of the history of different countries’ competitiveness. Unlike the classical methods that count on the statistics of various economic data, we investigate the dynamical paths of the countries’ competitiveness and the export basket based on the state of the art FCM method and the Complex Index of Relative Development (CIRD) [9]. We then build up the interdependency relationship between products that determines whether a product depends on other products. A country could benefit from the product relationship when evaluating its ability to develop some new products. Additionally, we investigate the economic similarity between countries. When a developing country designs economic strategies, it could use the experience of particular developed countries that have similar development history. The proposed method is applied to the international trade networks, which could recommend suitable developed countries to developing countries.

## 2. Results

In the section, we start by investigating the origin of the “middle-income trap” in Section 4.1. In Section 4.1, the product dependency is proposed to characterize the problem of how successful countries upgrade their industrial structure from low- to high-complexity products, where the product complexity is characterized by the Fitness-Complexity Method (FCM) [3]. In Section 4.2, a novel method is proposed to recommend developed countries for developing countries to learn from their historical development. The experimental observations are shown in Section 4.3.

In the experiments, we used international trade data from 1962 to 2000 to analyze the evolving paths of the fitness of countries and the Complex Index of Relative Development (CIRD) (see Section 4 for more details). The fitness is an effective index to characterize the competitiveness of countries, which has been utilized to forecast the economy, analyze scientific collaboration network, etc. [3,24] (see the Section 4.2). Developed (Developing) countries usually have high (small) fitness. CIRD is a hybrid index that combines fitness and the GDP per capita growth rate of country. CIRD outperforms fitness in predicting the development paths of countries [9] (see Section 4.3).

### 2.1. Interdependency between Products

Poor developing countries usually have small basket of products and the exported products are of low-complexity. With the economic growth, they raise some economic policies to upgrade their industrial structure. However, only a small number of countries successfully grow into developed countries. The reason is that the goals of many successful countries are overly ambitious and they have no potential to produce some high-complexity products. Consequently, the central problem is how to evaluate the potential of a country to develop a new certain product.

The potential of countries could be obtained based on the development history of countries. Historically, successful countries develop products from low- to high-complexity consecutively. We define a matrix $Y=(Y_{c,i})$, where $Y_{c,i}$ means the year that country *c* starts to export product *i*. If country *c* does not export product *i*, we set $Y_{c,i}=+\infty$. For two different products *i* and *j*, if a country *c* started to produce *i* at the year $Y_{c,i}$ and later produced *j* at the year $Y_{c,j}$ ($Y_{c,i}<Y_{c,j}$), we say that product *i* is the primary base of *j*. The dependency of product *j* on *i* is characterized by average number of years passing between the introduction of product *i* and the introduction of product *j* in the same country,

(1) $$d_{j,i}=\frac{1}{N_{i,j}}\sum_{Y_{c,i}\neq +\infty ,Y_{c,j}\neq +\infty }\left(Y_{c,j}-Y_{c,i}\right),$$

where $N_{i,j}$ is the number of countries who export products *i* and *j* simultaneously. We only count countries that produce the two products *i* and *j* at the same time. Note that, large $d_{j,i}$ means that the two products *i* and *j* have large time intervals when a country introduces the two products, implying weak relationship. Consequently, smaller $d_{j,i}$ means stronger dependency. For example, South Korea started to export fish many years ago and began to export competitive electric products in 1978. Fish and electric products have very large $d_{j,i}$ and little relationship. However, South Korea started to export DRAM memory chips in about 1980 and Mobile phones in about 1990. DRAM memory chips and Mobile phones have small $d_{j,i}$ and strong correlation. Thus, we can utilize $d_{j,i}$ to evaluate the dependency between products. Besides, notice that $d_{j,i}<0$ meaning product *j* is easier than *i* and a country exports product *j* earlier than *i* on the whole.

We note that, apart from Equation (1), Zaccaria et al. [25] categorized the products based on the frequency of occurrence of pairwise products, where the occurrence means that a country produces the two products simultaneously. They use the product taxonomy to recommend new products for countries, with satisfying accuracy. However, Equation (1) analyzes the product interdependency from the perspective of time intervals regardless of the frequency of occurrence of pairwise products.

### 2.2. The Economic Similarity between Countries

The potential of developing countries for a new product could be evaluated by the product dependency. For a certain complex product, if a countries has exported all the primary products, it has the potential to develop the complex product, which is also discussed in Ref. [25]. At the country level, a more general problem is how to design economic policies for deciders. Here, we suppose that developing countries could learn from the past of developed countries with a similar development history. The central idea is to the characterize the similarity between countries. The economic history could be described from various backgrounds, such as GDP per capital growth rate, education capacity, physical investment, and so on. Since CIRD provides a convenient index to evaluate the development of countries, we mainly investigate the CIRD similarity of different countries that is characterized by the Pearson correlation. Since different countries develop in different stages, we add offset to the CIRD vectors, where every CIRD vector represents a country’s CIRD history,

(2) $$r_{c_{1},c_{2}}\left(n\right)=Pearson\left(CIRD_{c_{1}}\left(n\right),CIRD_{c_{2}}\left(0\right)\right),$$

where $CIRD_{c_{1}}\left(0\right)=\left[CIRD_{c_{1},1},CIRD_{c_{1},2},\ldots ,CIRD_{c_{1},n}\right]$ means the original CIRD history of the country $c_{1}$, and $CIRD_{c_{1}}\left(n\right)$ means that we shift all the elements of $CIRD_{c_{1}}\left(0\right)$ to the left by length *n* and fill 0 in the right empty entries. For example, if $CIRD_{c_{1}}\left(0\right)=\left[0.1,0.3,0.5\right]$ and $CIRD_{c_{1}}\left(2\right)=\left[0.5,0,0\right]$. We can calculate the similarity $r_{c_{1},c_{2}}\left(n\right)$ with various *n* and recommend the countries with high similarity scores for a certain developing one. After shifting the CIRD vector, the Pearson correlation could reflect how many years a country falls behind another one, since different countries develop at different levels.

The fitness is an effective index to evaluate the competitiveness of a country. To improve the competitiveness, a country could also learn the experience from some developed countries with the past evolution of fitness,

(3) $$r_{c_{1},c_{2}}^{\prime }\left(n\right)=Pearson\left(F_{c_{1}}\left(n\right),F_{c_{2}}\left(0\right)\right),$$

where $F_{c_{1}}\left(0\right)=\left[F_{c_{1},1},F_{c_{1},2},\ldots ,F_{c_{1},n}\right]$ is the fitness history of the country $c_{1}$ and $F_{c_{1}}\left(n\right)$ means adding offset to the vector as well as $CIRD_{c_{1}}\left(n\right)$.

Note that both $r_{c_{1},c_{2}}\left(n\right)$ and $r_{c_{1},c_{2}}^{\prime }\left(n\right)$ could help developing countries to choose appropriate developed countries. We compare their performances in the Section 2.3.

### 2.3. Experimental Results

In the experiments, when calculating the fitness of countries and complexity of products, we set the initial $F_{i}^{\left(0\right)}=1$ and $Q_{\alpha }^{\left(0\right)}=1$ and iterated Equations (5) and (6) until the rankings of countries and products did not change. The CIRD for different countries at different years is published in [9].

#### 2.3.1. “Middle-Income Trap” on Product Interdependency

**Figure 1:**
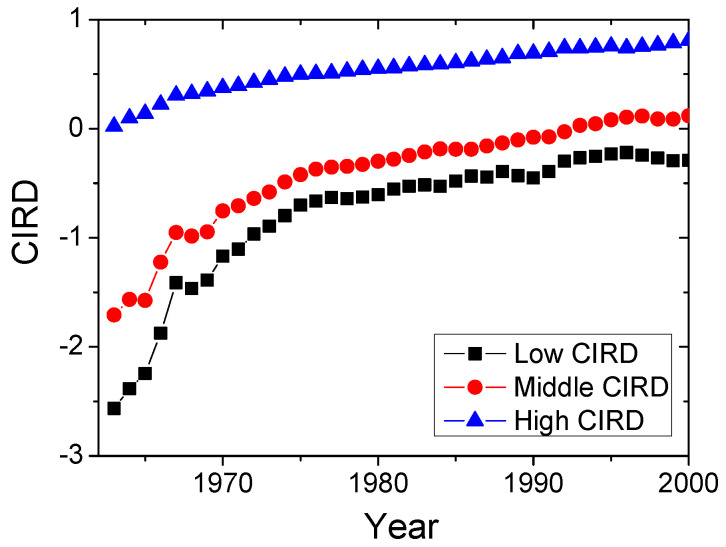
(Color online) The evolving paths of average CIRDs for different kinds of countries. We divide all countries into three different categories: The top 1/3 CIRD countries are considered as developed countries. The bottom 1/3 CIRD countries are developing countries. The other 1/3 countries are middle countries. We then calculate the average CIRD for different groups of countries.

We first investigated the evolving paths characterized by CIRD (Figure 1). We divided all countries into three different categories from high CIRD to low CIRD. For high CIRD countries, the average CIRD increase slowly, indicating the small GDP per capital growth rate, whereas, for low CIRD countries, the average CIRDs first increase sharply before 1970, and then increases smoothly. CIRD≈−2 is the flex point that has the largest increase of CIRD and means the start of the take-off stage (see more analysis of CIRD≈−2 in Ref. [9]).

**Figure 2:**
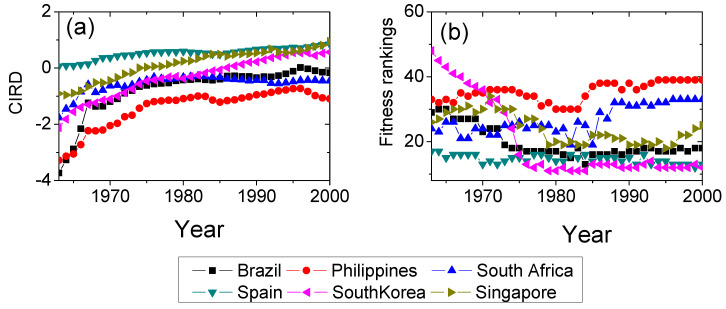
(Color online) The evolving paths of CIRD and fitness rankings for different countries. Brazil, Philippines and South Africa locate in the “middle-income trap”, while Spain, South Korea and Singapore successfully stepped out of the “middle-income trap” and became developed countries. (**a**) The CIRD evolving paths for the six countries. (**b**) The fitness rankings for the six countries. We sort the countries by the descending order of fitness, thus smaller ranking means higher fitness.

In this study, we were particularly interested in how to escape from the “middle-income trap”. The first step was to investigate the characteristics of both successful and unsuccessful countries. Figure 2 shows the evolving paths of CIRD and fitness rankings for successful countries (Spain, South Korea and Singapore) and unsuccessful countries (Brazil, Philippines and South Africa). Spain is a classical developed country, while before 1970, South Korea and Singapore were developing countries. In Figure 2a, we see that the CIRDs of South Korea and Singapore increase and approach Spain with high speed, whereas, for the unsuccessful countries, although their CIRDs increase sharply before 1970, the CIRDs increase slowly or even keep stable, falling into the “middle-income trap” after 1970. Besides CIRD, the fitness rankings can also reflect the development of countries (see Figure 2b). In Figure 2b, successful countries increase their fitness with small rankings, while unsuccessful countries have large fitness rankings, except Brazil. Brazil has ranked higher than Singapore after 1985 overall. However, Brazil is not a developed country. Brazil is a much larger country than the other countries and larger countries are more likely to have the ability to produce complex products. According to the fitness theory, diversifying the export basket could increase the fitness of countries. Thus, fitness fails in characterizing the development of Brazil and CIRD is better for the case.

**Figure 3:**
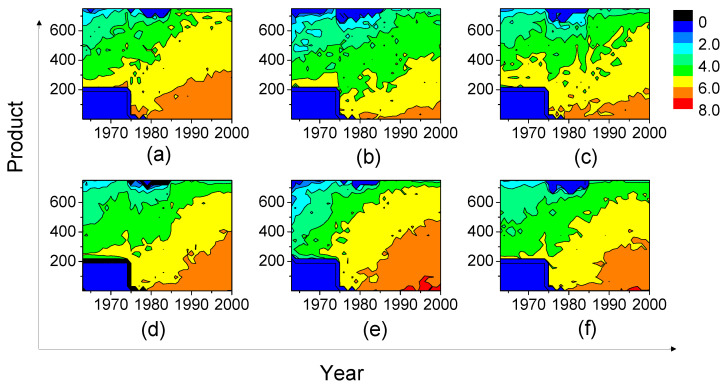
(Color online) The detailed export volumes of different products for different countries: (**a**) Brazil; (**b**) Philippines; (**c**) South Africa; (**d**) Spain; (**e**) South Korea; and (**f**) Singapore. The color depth at point (x,y) indicates the logarithm of the RCA of product *y* in the year *x*. If RCA=0, we set the logarithm of RCA as −1000. In the *y*-axis, the products are sorted by the ascending order of their complexity $Q_{i}$ in the year 2000. The *x*-axis is the different years from 1962 to 2000. Note that in the left-right area of the six panels, the RCA for the products with orders 1–210 are set to 0 due to the missing of the original data, which does not influence the analysis of the results.

To understand the influence of “middle-income trap”, we investigate the detailed export volumes (characterized by RCA) of different products for different countries in Figure 3. We see that the developed countries export more high-complexity products on the whole except Brazil. As one of the “BRICS” countries (Brazil, Russia, India, China, South Africa), Brazil exports diverse products and thus has the potential to become part of the developed countries. We see in Figure 3 that developed countries export much more primary low-complexity products than developing countries, which violates the economic theory of Ricardo that emphasizes the importance of high-complexity products for developed countries. We note that developed countries export a large quantity of low-complexity products as well as high-complexity products. This finding agrees with recent research that countries should diverse their baskets of export products [3,7], which also emphasizes the importance of low-complexity products as well as high-complexity products.

**Figure 4:**
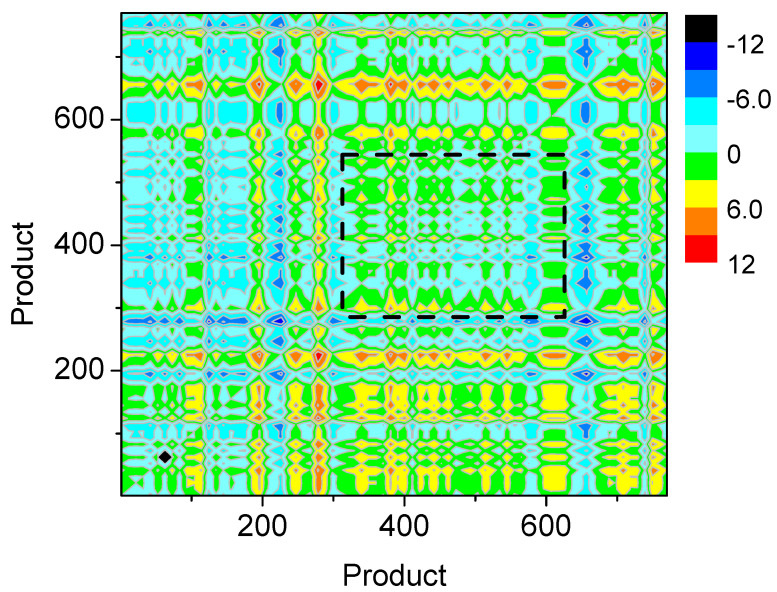
(Color online) The dependency of products on each other. The color depth of a point (i,j) indicates the dependency $d_{i,j}$ of product *j* on *i*. Green color means countries introduce the two products simultaneously (or with very short time intervals), implying strong interdependence. Red color means long time intervals exist when countries introduce the two products, indicating weak interdependence. In the panel, the products are sorted by the ascending order of their complexity $Q_{i}$ in 2000. Note that the rectangle area of the dotted line means products in the areas have low $d_{i,j}$ and high interdependency.

**Figure 5:**
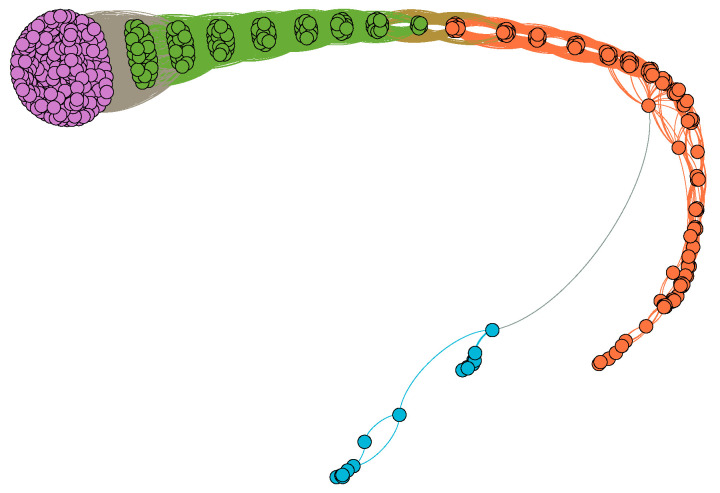
(Color online) The community structure of product relationship. The network $G=(g_{i,j})$ of product relationship is constructed by binarizing the matrix Y: if $|Y_{i,j}|<0.1$, $g_{i,j}=1$; otherwise, $g_{i,j}=0$. Besides, we do not consider the case $Y_{i,j}=0$ because no country introduces the two products *i* and *j* during 1962–2000. The different colors represent different communities that are calculated in Ref. [26]. The purple nodes, green nodes and yellow nodes are low-complexity, middle-complexity and high-complexity products, respectively. Nodes in the same communities have strong interdependence between themselves.

We further explored the difficulty for a country to develop new products based on the interdependency of products in Equation (1) (see Figure 4). The dependency of product *j* on product *i* describes the average number of years passing between the introduction of product *i* and the introduction of product *j* in the same country. Smaller time intervals between two products (smaller $d_{j,i}$) means stronger interdependence, while longer time intervals between two products (higher $d_{j,i}$) means weaker interdependence. In Figure 4, low-complexity products rarely depend on high-complexity products. Interestingly, some high-complexity products depend little on other products. The reason is that they are innovative products (e.g., mobile phones and unmanned aerial vehicles). Developed countries also have large time intervals between the innovative products and the previous industrial products. Additionally, we show that a large number of middle-complexity products depend much on each other, as shown in the rectangle area with the dotted line in Figure 4. Figure 5 clearly shows the relationship between products. In Figure 5, products could be divided into some clusters. Nodes in the same clusters have strong interdependency between themselves. It indicates that, when upgrading their industrial structure, developing countries should develop most of the products in the area simultaneously (or easy access to these products would be prohibitively expensive due to intellectual property protection and other problems). Introducing a large number of new products in the rectangle area at the same time is difficult for developing countries, which means the high difficulty for upgrading industrial structure. Consequently, many developing countries are likely to fall into the “middle-income trap”, as described in the Section 1.

#### 2.3.2. Economic Similarity Based on CIRD and FCM

**Figure 6:**
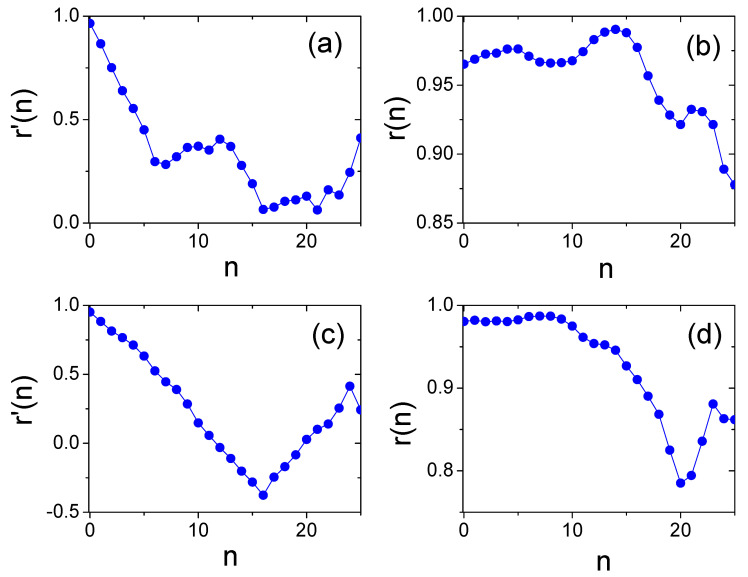
(Color online) (**a**) The Pearson correlation $r^{\prime }\left(n\right)$ of Equation (3) between South Korea and Dominican Republic. (**b**) The Pearson correlation r(n) of Equation (2) between South Korea and Dominican Republic. (**c**) The Pearson correlation $r^{\prime }\left(n\right)$ of Equation (3) between Singapore and Malaysia. (**d**) The Pearson correlation r(n) of Equation (2) between Singapore and Malaysia.

Another issue is that developing countries usually face the problem of how to design effective economic policies. A possible solution is to follow developed countries with similar development history. Taking South Korea and Singapore as an example, Figure 6 shows the Pearson correlation between South Korea and Dominican Republic, and the correlation between Singapore and Malaysia based on the fitness and CIRD. High correlation means high similarity between the two countries. We found that, for the fitness of countries, $r^{\prime }\left(0\right)$ reaches maximum in more than four countries, while the correlation based on CIRD could reflect how many years a country develops behind another country. After traversing the similarity between the targeted country and the other countries for different time offset *n*, we can recommend the optimal country with the maximum r(n) to the targeted country. Table 1 lists the recommended countries to the targeted ones for the “BRICS” countries plus Philippines, since Philippines has a promising development according to the fitness of countries in Refs. [3,27]. The recommendation list by r(n) seems more reasonable in practice. We argue that the recommendation list could supply additional information for developing countries.

**Figure 7:**
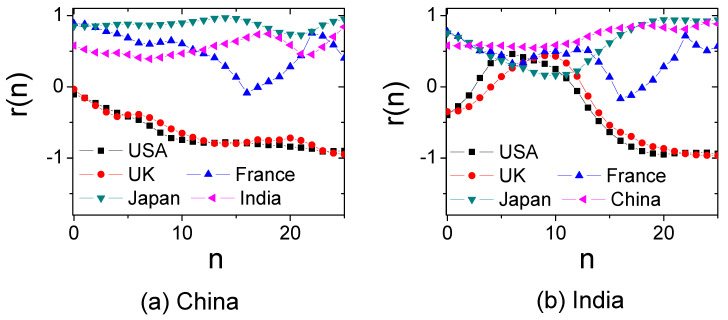
(Color online) (**a**) The Pearson correlation r(n) of Equation (2) between China and four traditional developed countries plus India. (**b**) The Pearson correlation r(n) of Equation (2) between India and some traditional developed countries plus China.

**Figure 8:**
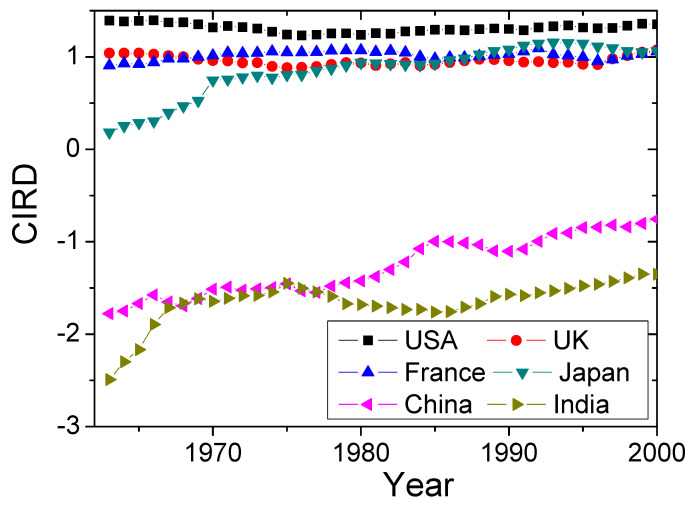
(Color online) The evolving paths of CIRD for China, India and four traditional developed countries.

Finally, we investigated the economics of China and India based on r(n), since the two countries have achieved incredible development in the last several decades and attract much attention. Figure 7 shows the Pearson correlation r(n) of Equation (2) between China, India and four traditional developed countries. We see that China and Japan have the highest similarity. Besides, Figure 7 shows that China is about 14 years behind Japan. This is in agreement with the Human Development Index in Human Development Reports (http://hdr.undp.org/en/data), stating that China requires about 10–15 years to reach the current level of Japan. It also explains why China follows a similar economic development to Japan in many fields. India is similar to both Japan and China, yet with about 20 years delay with the two countries. In practice, India also cooperates much with Japan. Moreover, India is more similar to China than to Japan, which is reflected by the CIRD dynamics in Figure 8. However, the Pearson correlation r(n) cannot capture the economic trend. Integrating Figure 7 and Figure 8, we see that India lags behind China, yet has the same CIRD level as China. Hence, China could also supply much economic experience to India.

## 3. Discussion

The problem of middle-income trap has been widely discussed and investigated by using the detailed economic data of countries [28,29,30]. The results based on detailed data could provide valuable advice for developing countries. However, most of the analysis only emphasizes the importance of the high-complexity products. Actually, upgrading the industrial structure from low- to high-complexity products could make more profits for the countries. We note that it does not mean we should discriminate (or reduce) the industrial structure of low-complexity products, since developed countries export more low-complexity products in terms of RCA [30].

The previous network-based work on FCM gave an in-depth explanation of how the product basket of countries and the fitness relate with future growth. It is not clear however how to increase the fitness itself, and how to help countries move outside of the trap. Here, we propose the product dependency that is rarely considered in previous statistics. Our coarse product dependency provides a different solution to the origin of the middle-income trap, i.e., why there is a barrier in the fitness itself and why it is difficult to diversify the export basket from basic commodities to high-tech products. We then investigate the economic similarity between countries for developing countries. The experience of successful countries that get through the middle-income trap may inspire the current developing ones. Our method could help developing countries find the most similar developed countries to learn their economic policies.

Though our work provides a novel perspective to inspect the problem of middle-income trap, the precision is limited due to the lack of detailed data for every country [31]. However, our results could explain some confusing phenomena in the classical statistical methods. A possible extension of the work is to combine the results with traditional economic indices [29,30,32] to improve the precision.

## 4. Materials and Methods

### 4.1. Dataset Description

We used the international trade data from 1962 to 2000 [16,27,33]. The country–product relationship was represented by a bipartite network in which one kind of nodes indicate the countries and the other ones indicate the products. In the original international trade data, products are categorized into different classes. Since different countries may categorize a certain product into different classes, we cannot differentiate the complexity of a certain product only based on the data. Besides, we can only obtain a fraction of the countries’ products due to the incomplete database. Actually, only 72 countries reported their exports to the UN database. Please see Ref. [27] for the full procedure to process the original data. After filtering the data, the network contained 72 countries and 770 product categories.

Different countries export different baskets and quantities of products. Here, we take a simple approach and only consider the total export in US$ of products of a country. In practice, a country may produce more or less of a product. To characterize whether a country is a competitive exporter of a product, we use the “Revealed comparative advantage” (RCA) to re-normalize the weight of the country-product relations and only edges with weight larger than 1 are reserved. The RCA is defined as

(4) $$RCA_{i\alpha }=\frac{e_{i\alpha }/\sum_{j}e_{j\alpha }}{\sum_{\beta }e_{i\beta }/\sum_{j\beta }e_{j\beta }},$$

where $e_{i\alpha }$ is the export in US$ of country *i* for product α. After processing the data by RCA, we obtained the country-product bipartite weighted network, denoted by $M={\left(M_{ij}\right)}_{N_{c}\times N_{p}}$, where $N_{c}$ and $N_{p}$ represent the size of countries and products, respectively.

Apart from the international trade network from 1962 to 2000, we also investigated the trade network from 1998 to 2014 (see the data in Refs. [27,34]). Since the result is similar to the 1962–2000 data, we only discuss the the 1962–2000 data in the paper.

### 4.2. Fitness-Complexity (FCM)

Fitness-Complexity Method (FCM) defines the country fitness $\left\{F_{i}\right\}$ and product complexity $\left\{Q_{\alpha }\right\}$ as the stationary point of the following nonlinear recursive process [3],

(5) $$\begin{array}{c} {\tilde{F}}_{i}^{\left(n\right)}=\sum_{\alpha }M_{i\alpha }Q_{\alpha }^{\left(n-1\right)},\\ {\tilde{Q}}_{\alpha }^{\left(n\right)}=\frac{1}{\sum_{i}M_{i\alpha }\frac{1}{F_{i}^{\left(n-1\right)}}}, \end{array}$$

where the scores are normalized after each step by

(6) $$\begin{array}{c} F_{i}^{\left(n\right)}={\tilde{F}}_{i}^{\left(n\right)}/<{\tilde{F}}_{i}^{\left(n\right)}>,\\ Q_{\alpha }^{\left(n\right)}={\tilde{Q}}_{\alpha }^{\left(n\right)}/<{\tilde{Q}}_{\alpha }^{\left(n\right)}>, \end{array}$$

where <…> is the average operation. The initial values of $F_{i}^{\left(0\right)}$ and $Q_{\alpha }^{\left(0\right)}$ do not influence the final stationary state except some particular singular points. Without loss of generality, we set the initial condition $F_{i}^{\left(0\right)}=1$ and $Q_{\alpha }^{\left(0\right)}=1$ and iterated Equations (5) and (6) until the rankings of countries and products did not change (see [35] for the convergence of the iteration). When we applied Equation (5) to real country–product bipartite networks, developed countries and high-complexity products tended to have large final values, while developing countries and low-complexity products tended to have small final values. Therefore, FCM could evaluate the competitiveness of countries and the complexity of products [3,36], and predict the future economic development [27,37]. The index of FCM outperforms the degree-based index [7]. Actually, FCM is a famous variant of the primary method of reflections, as discussed in [19,38]. Besides, FCM has also been applied to other fields, such as ecological networks [39] and scientific competitiveness of nations [24].

### 4.3. The Complex Index of Relative Development (CIRD)

In the economic analysis, an important task is to predict the future growth of a country, which is often evaluated by the GDP per capita or average wage. Empirical successful countries, such as Japan, Southern Europe, and the Asian Tigers, have experienced a decade or more of extremely high growth which is characterized by a strong increase in investments, in both physical and human capital [9]. Since the GDP per capita growth rate is influenced by various factors, we should investigate how the GDP growth is influenced by different factors.In the classical method, the GDP per capita growth rate $y_{c,t}$ is described as [9,40]

(7) $$y_{c,t}=a_{c,t}+\alpha k_{c,t}+\left(1-\alpha \right)e_{c,t}+\left(1-\alpha \right)h_{c,t},$$

where $a_{c,t}$ is the growth rate contribution of the exogenous technological efficiency of the country *c* at time *t*, αk is the growth contribution of physical capital, (1−α)e is the growth contribution of the labor force share in population, (1−α)h is the growth contribution of the human capital (education) of workers of the country, and α is the output elasticity of capital. Some previous studies [9,40] provide a general way to decompose the influence of different factors.

According to Equation (7), a country will linearly get high GDP per capita growth rate with the input investments. However, when the investment is large, due to the limitation of various input factors, GDP per capita growth rate cannot be improved by only increasing the investments. For example, a country could double its labor force share in population from 10% to 20%, yet cannot double the factor when the share reaches 60%. Thus, GDP per capita growth rate is very large for many countries during the take-off stage, but declines when the industrialization of the country finishes. Consequently, developing countries usually have large growth rate, while developed countries have small or even negative growth rate. However, Equation (7) cannot reflect this nonlinear phenomenon.

Based on the fitness of countries in Equation (5), Emanuele et al. [9] proposed a hybrid index to evaluate the growth of countries, i.e., the Complex Index of Relative Development (CIRD),

(8) $$\begin{array}{cc} CIRD_{c,t} & =log(F_{c,t}^{\beta }GDPpc_{c,t}^{1-\beta })\\ & =\beta log\left(F_{c,t}\right)+\left(1-\beta \right)log\left(GDPpc_{c,t}\right), \end{array}$$

where $F_{c,t}$ and $GDPpc_{c,t}$ represent the fitness and GDP per capita growth rate of country *c* at time *t*, respectively. β is a tunable parameter to balance the $F_{c,t}$ and $GDPpc_{c,t}$. Pugliese et al. [9] used CIRD with tunable β to investigate the development of countries. When β=0.18, $CIRD_{\left(c,t\right)}$ performs well in real data. Thus, we set β=0.18 in the experiments.

Since CIRD contains the information of both $F_{c,t}$ and $GDPpc_{c,t}$, we can explain the development of a country from multiple perspectives. On the one hand, for developing countries, increasing the domestic investments results in the increase of both $F_{c,t}$ and $GDPpc_{c,t}$, meaning high growth of CIRD, whereas, for developed countries, both $F_{c,t}$ and $GDPpc_{c,t}$ fluctuate a little and CIRD increases slowly. On the other hand, CIRD could also predict the development of different countries. To increase CIRD, we can increase $F_{c,t}$ or $GDPpc_{c,t}$, or both. Increasing $F_{c,t}$ means that a country should diversify their basket of products and produce some high-complexity products, while increasing $GDPpc_{c,t}$ requires a country to export more products. According to the analysis of empirical data, a developing country should first diversify their basket of products and then improve the complexity of their products [3,37]. Comparing with the GDP per capita growth rate, CIRD is a better index to evaluate the development of a country [9]. Before the take-off stage of a country, the GDP per capita growth rate is small, while CIRD may be large due to the diverse export baskets, such as in China and India.

## 5. Conclusions

In summary, we investigate the development history of countries in terms of FCM and CIRD. We show that developed countries export more low-complexity products than developing countries, indicating that, when a developing country gets through the middle-income trap, it should develop low-complexity products as well as high-complexity products. Based on the economics of different countries, we then build up the interdependency relationship between products. Interestingly, some middle-complexity products have strong inner correlation, which increases the difficulty of developing countries when upgrading the industrial structure, since middle-complexity products should be developed simultaneously. Moreover, we investigate the economic similarity between countries. The experience of successful countries that get through the middle-income trap may inspire the current developing countries. Our method could help developing countries find the most similar developed countries to learn their economic policies. Therefore, our work could supply additional information that organizations could benefit from by considering our analysis.

## Figures and Tables

**Table 1:** The recommended countries based on correlations r(n) and $r^{\prime }\left(n\right)$. Here, only the countries with the largest scores r(n) and $r^{\prime }\left(n\right)$ are recommended to the targeted countries.

Target Country	Recommended Country Based on r(n)	Recommended Country Based on $r^{\prime }\left(n\right)$
Brazil	New Zealand	Israel
Russia	Romania	Saudi Arabia
India	South Korea	Peru
China	Finland	Indonesia
Pilipinas	Thailand	Israel
